# Smoking Status and Type 2 Diabetes, and Cardiovascular Disease: A Comprehensive Analysis of Shared Genetic Etiology and Causal Relationship

**DOI:** 10.3389/fendo.2022.809445

**Published:** 2022-02-18

**Authors:** Yanna Chi, Xinpei Wang, Jinzhu Jia, Tao Huang

**Affiliations:** ^1^ Department of Biostatistics, School of Public Health, Peking University, Beijing, China; ^2^ Center for Statistical Science, Peking University, Beijing, China; ^3^ Department of Epidemiology & Biostatistics, School of Public Health, Peking University, Beijing, China; ^4^ Department of Global Health, School of Public Health, Peking University, Beijing, China; ^5^ Key Laboratory of Molecular Cardiovascular Sciences (Peking University), Ministry of Education, Beijing, China

**Keywords:** smoking status, type 2 diabetes, cardiovascular disease, shared genetic etiology, causality

## Abstract

**Objective:**

This study aimed to explore shared genetic etiology and the causality between smoking status and type 2 diabetes (T2D), cardiovascular diseases (CVDs), and related metabolic traits.

**Methods:**

Using summary statistics from publicly available genome-wide association studies (GWASs), we estimated genetic correlations between smoking status and T2D, 6 major CVDs, and 8 related metabolic traits with linkage disequilibrium score regression (LDSC) analysis; identified shared genetic loci with large-scale genome-wide cross-trait meta-analysis; explored potential shared biological mechanisms with a series of post-GWAS analyses; and determined causality with Mendelian randomization (MR).

**Results:**

We found significant positive genetic associations with smoking status for T2D (Rg = 0.170, *p* = 9.39 × 10^−22^), coronary artery disease (CAD) (Rg = 0.234, *p* = 1.96 × 10^−27^), myocardial infarction (MI) (Rg = 0.226, *p* = 1.08 × 10^−17^), and heart failure (HF) (Rg = 0.276, *p* = 8.43 × 10^−20^). Cross-trait meta-analysis and transcriptome-wide association analysis of smoking status identified 210 loci (32 novel loci) and 354 gene–tissue pairs jointly associated with T2D, 63 loci (12 novel loci) and 37 gene–tissue pairs with CAD, 38 loci (6 novel loci) and 17 gene–tissue pairs with MI, and 28 loci (3 novel loci) and one gene–tissue pair with HF. The shared loci were enriched in the exo-/endocrine, cardiovascular, nervous, digestive, and genital systems. Furthermore, we observed that smoking status was causally related to a higher risk of T2D (β = 0.385, *p* = 3.31 × 10^−3^), CAD (β = 0.670, *p* = 7.86 × 10^−11^), MI (β = 0.725, *p* = 2.32 × 10^−9^), and HF (β = 0.520, *p* = 1.53 × 10^−6^).

**Conclusions:**

Our findings provide strong evidence on shared genetic etiology and causal associations between smoking status and T2D, CAD, MI, and HF, underscoring the potential shared biological mechanisms underlying the link between smoking and T2D and CVDs. This work opens up a new way of more effective and timely prevention of smoking-related T2D and CVDs.

## Introduction

Despite concerted efforts to combat the global tobacco epidemic, tobacco smoking remains the leading preventable cause of morbidity and mortality (1). Smoking has multiple well-known adverse health effects (2, 3), and its association with type 2 diabetes (T2D) and cardiovascular diseases (CVDs) has been a major public health concern. Considerable studies, both prospective cohort studies among different population groups (4–6) and meta-analyses (7–10), have provided compelling evidence of the important role of smoking in increasing the risk of T2D and CVDs. Approximately 30%~40% of the increased risk of T2D (2) and 20%~30% of all CVD deaths (11, 12) compared to never smokers are attributed to smoking. In addition, previous twin or family studies have shown that smoking, T2D, and many CVDs, such as coronary artery disease (CAD), are heritable traits (13–15), and the heritability was estimated to range from 4% to 19% for smoking phenotypes (16, 17), 17%~23% for T2D (18), and 14%~21% for CAD (19, 20) in recent large-scale genome-wide association studies (GWASs). Furthermore, genetic correlations between several smoking phenotypes and T2D or CVDs have been observed (16, 21). For example, two recent large-scale GWASs on tobacco use revealed that smoking initiation was genetically positively correlated with T2D, CAD, myocardial infarction (MI), and heart failure (HF) and that cigarettes per day and smoking cessation were genetically positively correlated with CAD. More interestingly, single-nucleotide polymorphisms (SNPs) in some genes have been reported to have effects on both smoking and T2D or CVDs (22–24).

These lines of evidence suggest two possibilities to account for such associations between smoking and T2D or CVDs. One is pleiotropy. Smoking and T2D or CVDs may share common genetic variants that simultaneously influence two or more of these traits or disorders by engaging in common pathways or controlling common risk factors. An alternative possibility is that causal associations may exist between smoking and T2D or CVDs. In recent years, large publicly available GWAS datasets and multiple state-of-the-art statistical analysis methods including linkage disequilibrium score regression (LDSC) (25), cross-trait meta-analysis (26), transcriptome-wide association studies (TWAS) (27), and Mendelian randomization (MR) analysis (28–31), can be utilized to facilitate investigations of whether the comorbidity and risk interrelationship of these traits or disorders can be explained by common genetic variants or causality. Given these possibilities and methodological advances, it is now important and feasible for us to elucidate the mechanisms underlying the comorbidity between smoking and T2D and CVDs. As is apparent from the literature, the associations between smoking and T2D or CVDs varied due to the differences in the measurement of smoking in different studies (3–5, 8). In our study, we chose smoking status, an ordinal categorical variable, which is divided into current smokers, former smokers, and never smokers according to smoking intensity and recency.

To our knowledge, no genetic study has systematically explored the common genetic etiology between smoking status and T2D and CVDs. Therefore, in the present study, we conducted a comprehensive analysis using summary statistics from publicly available GWASs to explore shared genetic etiology and the causality between smoking status and T2D, CVDs, and related metabolic traits.

## Materials and Methods

### Study Design and Data Summary

**Figure 1:**
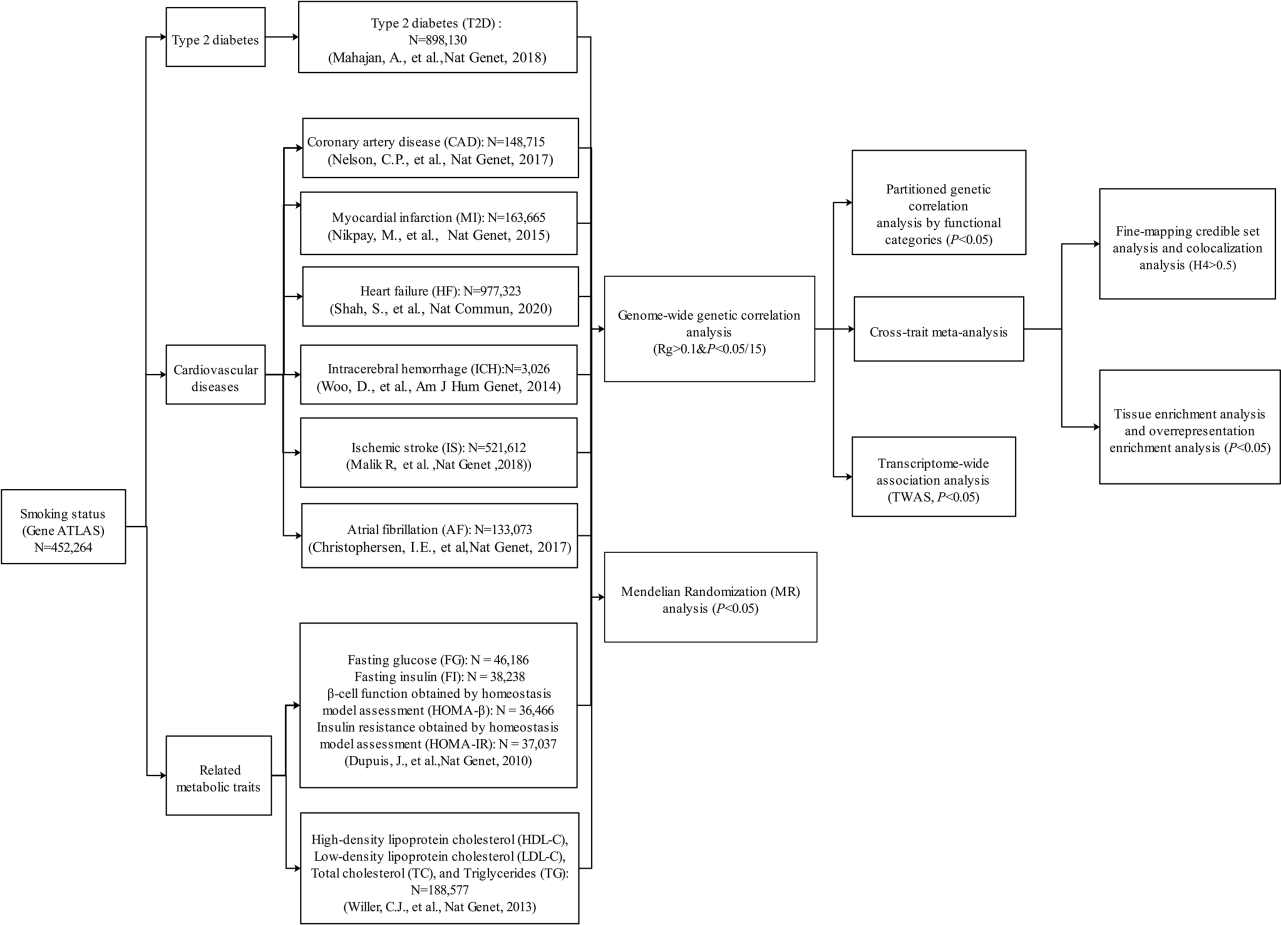
Overall study design. Multiple genome-wide association study (GWAS) data sources were first retrieved. We first conducted a genome-wide genetic correlation analysis between smoking status and type 2 diabetes (T2D), six cardiovascular diseases (CVDs), and eight related metabolic traits. For the traits that showed significant genetic correlation with smoking status, we further conducted post-GWAS analyses to investigate the genetic overlap between them (variant/region/functional levels). Then, we also explored the causal relationship between smoking status and T2D, six CVDs, and eight related metabolic traits.

The whole study design is shown in **Figure 1**. Summary statistics used in this study were extracted from publicly available GWASs. The dataset of smoking status was from Gene ATLAS, consisting of 452,264 participants (32, 33). We retrieved summary statistics from the Diabetes Genetics Replication And Meta-analysis (DIAGRAM) Consortium for T2D (N = 898,130) (18). Generally, CVDs encompass a broad range of disorders of the heart and blood vessels including coronary heart disease, cerebrovascular disease, and other conditions. In this study, we chose six common or devastating CVDs including CAD (N = 148,715) (20) and MI (N = 163,665) (34) from the Coronary Artery Disease Genome wide Replication and Meta-analysis (CARDIoGRAM) plus the Coronary Artery Disease (C4D) Genetics (CARDIoGRAMplusC4D) consortium, HF (N = 977,323) (35) from the Heart Failure Molecular Epidemiology for Therapeutic Targets (HERMES), ischemic stroke (IS; N = 521,612) from the METASTROKE collaboration (36), intracerebral hemorrhage (ICH; N = 3,026) from the International Stroke Genetics Consortium (37), and atrial fibrillation (AF; N = 133,073) from the Atrial Fibrillation Genetics Consortium (38). In addition, several important T2D/CVD-related metabolic traits were considered in this study, including glycemic traits [fasting glucose (FG; N = 46,186), fasting insulin (FI; N = 38,238), and the surrogate estimates of β-cell function (HOMA-β; N = 36,466) and insulin resistance (HOMA-IR; N = 37,037) derived from fasting variables by homeostasis model assessment from the Meta-Analyses of Glucose and Insulin-related traits Consortium (39) and blood lipids [high-density lipoprotein cholesterol (HDL-C), low-density lipoprotein cholesterol (LDL-C), total cholesterol (TC), and triglyceride (TG), N = 188,577] from the Global Lipids Genetics Consortium (40). The majority of the participants were of European ancestry in each GWAS (**Supplementary Table 1**). Detailed disease definition and baseline characteristics for each study were described in previous studies (18, 20, 32–40). For example, smoking status, an ordinal categorical variable based on several questions about smoking intensity and recency, includes the categories of current smokers (those who have smoked 100 cigarettes in their lifetime and currently smoke cigarettes), former smokers (those who have smoked at least 100 cigarettes in their lifetime but had quit smoking at the time of interview), and never smokers (those who have never smoked or who have smoked less than 100 cigarettes in their lifetime) (32, 33). T2D status was defined based on multiple sources of evidence, including a self-reported history of T2D, doctor-diagnosed T2D, antidiabetic treatment, fasting plasma glucose >7.0 mmol/L, or 2-h plasma glucose >11.1 mmol/L (18). In CARDIoGRAMplusC4D, CAD status was defined by an inclusive CAD diagnosis, including MI, percutaneous transluminal coronary angioplasty (PTCA), coronary artery bypass grafting (CABG), chronic ischemic heart disease (IHD), and angina (20). More details of these datasets can be seen in the original publications or related websites (18, 20, 32–40). In this study, our analyses were restricted to autosomal chromosomes.

### Statistical Analysis

#### Linkage Disequilibrium Score Regression

We used LDSC, a method requiring only GWAS summary statistics and having no bias by sample overlap, to estimate genetic correlations between smoking status and T2D, 6 major CVDs, and 8 related metabolic traits (41). This method relies on an algorithm that multiplies the Z score of the same SNP and two different phenotypes and then regresses the product of the Z scores from two phenotypes on the LD that the SNP has with all neighboring SNPs (25, 41). The Bonferroni correction was used to adjust multiple testing (two-tailed *p* < 0.05/15).

#### Partitioned Genetic Correlation

Genetic correlations within functional categories between smoking status and T2D, CAD, MI, and HF were estimated using partitioned LDSC to further describe the genetic overlap at the level of functional categories (42). Eleven functional categories were involved, including the DNase I digital genomic footprinting (DGF) region, DNase I hypersensitivity sites (DHSs), fetal DHS, intron, super-enhancer, transcription factor-binding sites (TFBS), transcribed regions, and histone marks H3K4me1, H3K27ac, H3K4me3, and H3K9ac. This method recalculated LD scores for SNPs partitioned in each particular functional category to estimate the genetic correlation within that functional group.

#### Cross-Trait Meta-Analysis

We applied a cross-trait GWAS meta-analysis by the R package Cross-Phenotype Association (CPASSOC) to further identify shared loci of the above four trait pairs with strong and significant genetic correlation (26). This method is robust to sample overlap and accommodates different types of phenotypic traits, correlated, independent, continuous, or binary traits. In addition, the effects of trait heterogeneity, population structure, and cryptic relatedness can be controlled by CPASSOC (26). S_Het_ was chosen as the main statistics. SNPs with P_SHet_ < 5 × 10^−8^ and trait-specific *p* < 0.01 were considered to have effects on both traits.

#### Fine-Mapping Credible Set Analysis

To identify the regions of shared loci more precisely, fine-mapping credible set analysis based on a Bayesian algorithm was performed to determine credible sets of causal variants at each of the shared loci (43–45). The identified credible sets of causal variants were 99% likely to contain causal disease-associated SNPs by extracting variants that were highly linked (r^2^ > 0.4) with the index SNP and within 500 kb of the index SNP (46).

#### Colocalization Analysis

A colocalization analysis by the R package coloc was applied to determine whether the association signals of trait pairs colocalized at the same locus (47, 48). The probability that both traits are associated and share a single causal variant (Coloc H4 Prob) was calculated with variants extracted within 500 kb of the index SNP at each of the shared loci. Loci with Coloc H4 Prob greater than 0.5 were considered to colocalize (49).

#### Tissue Enrichment Analysis, Overrepresentation Enrichment Analysis, and Transcriptome−Wide Association Study Analysis

To further understand the biological insights of the identified shared genes between smoking status and T2D, CAD, MI, and HF, we conducted multiple post-GWAS functional analyses. Based on RNA-Seq data from the Human Protein Atlas (HPA) across 35 human tissues (50), we used the TissueEnrich web application to calculate the tissue-specific gene enrichment and further understand whether identified shared genes of each trait pair were enriched in disease-relevant tissues (51). We applied the WebGestalt application (52) to determine overrepresentation enrichment of the identified shared gene set in Gene Ontology (GO) biological processes (53, 54). Furthermore, we conducted TWAS using the FUSION software package and 48 Genotype-Tissue Expression (GTEx) (version 7) reference weights (27) to explore the gene expression association in different tissues between smoking status and T2D, CAD, MI, and HF. The false discovery rate (FDR) Benjamini–Hochberg procedure was applied to correct for multiple testing, and FDR < 0.05 was regarded as significant.

#### Bidirectional Mendelian Randomization Analysis

Finally, we used the TwoSampleMR package to perform a bidirectional MR analysis to explore the causality between smoking status and T2D, 6 major CVDs, and 8 related metabolic traits (28–31). Bidirectional MR is a form of causal inference analysis that can estimate causal directions and effects by employing genetic instruments selected from large-scale GWASs (55), even in the presence of unmeasured confounders. Three basic assumptions must be fulfilled to yield unbiased causal estimates in the MR analysis: 1) the genetic instruments used must be associated with the exposure, 2) the genetic instruments should be independent of the confounders between the exposure and outcome, and 3) the genetic instruments affect the outcome only through the exposure (46, 56). In this study, we extracted genetic instruments (SNPs) with *p* < 5 × 10^−8^ from the GWAS summary statistics of the exposure of interest, conducted the horizontal pleiotropy test, and selected independent genetic instruments at r^2^ < 0.001 to satisfy these three assumptions. For each potential causality, the inverse variance-weighted (IVW) method was used to obtain the primary causal estimates. The FDR Benjamini–Hochberg procedure was applied to correct for multiple testing (FDR < 0.05).

Notably, the T2D, CAD, and HF GWASs contained UK Biobank participants, which may overlap to some extent with smoking status GWAS from the UK Biobank. Therefore, we additionally extracted T2D, CAD, and HF GWAS summary statistics from earlier or smaller-scale GWASs (57–59) that did not contain UK Biobank participants to further confirm the potential causal associations between smoking status and T2D, CAD, and HF. The details of these GWASs are presented in **Supplementary Table 2**.

## Results

### Genome-Wide Genetic Correlation

Understanding the genetic correlations of different complex traits or diseases can provide preliminary insights into genetic etiology. Therefore, we firstly estimated genetic correlations between smoking status and T2D, 6 major CVDs, and 8 related metabolic traits by LDSC. Among these traits, T2D (Rg = 0.170, *p* = 9.39 × 10^−22^), CAD (Rg = 0.234, *p* = 1.96 × 10^−27^), MI (Rg = 0.226, *p* = 1.08 × 10^−17^), and HF (Rg = 0.276, *p* = 8.43 × 10^−20^) showed strong and significant positive genetic correlations with smoking status (**Table 1**). In addition, we found nominally significant positive genetic correlations with smoking status for IS, ICH, and FG (**Table 1**). Genetic correlations between smoking status and HDL-C or TG reached statistical significance, but the magnitude of genetic correlation was less than 10% (**Table 1**). However, we did not find evidence of genetic correlations with smoking status for AF, FI, HOMA-B, HOMA-IR, LDL-C, and TC (**Table 1**).

**Table 1:** Genetic correlations between smoking status and T2D, CVDs, and related metabolic traits (α = 0.05/15).

Phenotype 1	Phenotype 2		Rg	Rg_SE	*p*-Value
Smoking status	T2D		0.170	0.018	9.39E−22*
	CVDs	CAD	0.234	0.022	1.96E−27*
		MI	0.226	0.026	1.08E−17*
		HF	0.276	0.030	8.43E−20*
		IS	0.164	0.057	3.70E−03
		ICH	0.188	0.080	1.80E−02
		AF	0.029	0.029	3.17E−01
	Glycemic traits	FG	0.105	0.042	1.31E−02
		FI	0.048	0.055	3.84E−01
		HOMA−β	−0.012	0.052	8.13E−01
		HOMA−IR	0.064	0.058	2.72E−01
	Blood lipids	LDL−C	0.022	0.030	4.77E−01
		HDL−C	−0.094	0.024	6.14E−05
		TC	0.032	0.026	2.11E−01
		TG	0.096	0.026	2.00E−04

Rg, genetic correlation estimate; SE, standard error of genetic correlation estimate; T2D, type 2 diabetes; CAD, coronary artery disease; MI, myocardial infarction; HF, heart failure; IS, ischemic stroke; ICH, intracerebral hemorrhage; AF, atrial fibrillation; FG, fasting glucose; FI, fasting insulin; HOMA-β, β-cell function obtained by homeostasis model assessment; HOMA-IR, insulin resistance obtained by homeostasis model assessment; HDL-C, high-density lipoprotein cholesterol; LDL-C, low-density lipoprotein cholesterol; TC, total cholesterol; TG, triglyceride.

*A significant p-value after Bonferroni correction.

### Partitioned Genetic Correlation

**Figure 2:**
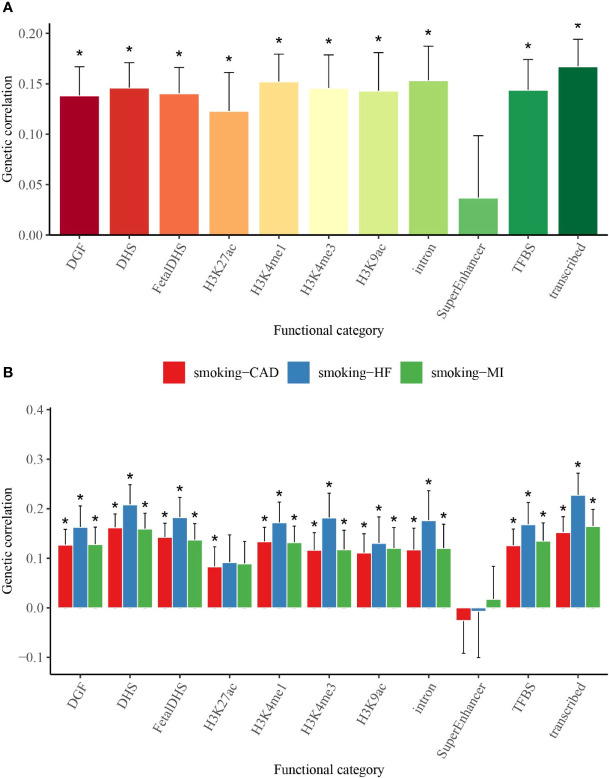
Partitioned genetic correlations of smoking status and T2D, CAD, MI, and HF. **(A)** Partitioned genetic correlations of smoking status and T2D. **(B)** Partitioned genetic correlations of smoking status and CAD, MI, and HF. The vertical axis represents the genetic correlation estimate; the horizontal axis represents 11 functional categories. The asterisk represents significance after Benjamini–Hochberg correction (FDR < 0.05); error bars represent the SE of the genetic correlation estimate. T2D, type 2 diabetes; CAD, coronary artery disease; MI, myocardial infarction; HF, heart failure; FDR, false discovery rate.

We used partitioned LDSC analysis to further evaluate genetic correlations between smoking status and T2D, CAD, MI, and HF in 11 functional annotations. Almost all the partitioned genetic correlations in each trait pair were positive (**Figure 2** and **Supplementary Table 3**). Large and statistically significant genetic correlations in many functional categories were observed, and a few categories stood out in particular. The highest magnitude of significant genetic correlation between smoking status and T2D (Rg = 0.167), MI (Rg = 0.164), and HF (Rg = 0.227) was in transcribed regions, where this region can transcribe DNA sequence to mRNA (**Figure 2** and **Supplementary Table 3**). Smoking status and CAD (Rg = 0.162) showed the highest magnitude of significant genetic correlation in DHSs, which are regions of chromatin that are sensitive to cleavage by the DNase I enzyme (**Figure 2** and **Supplementary Table 3**).

### Cross-Trait Meta-Analysis

The strong genetic correlations for smoking status and T2D, CAD, MI, and HF encouraged the exploration of common genetic architecture; therefore, we performed a genome-wide cross-trait meta-analysis to identify shared genetic loci between them (meta-analysis *p* < 5 × 10^− 8^; trait-specific *p* < 0.01). The lists of shared loci of each trait pair are provided in **Tables 2**, **3** and **Supplementary Tables 4–7**.

**Table 2:** Novel shared loci in the cross-trait meta-analysis of smoking status and T2D (*p*_meta_ < 5 × 10^−^8; single trait *p* < 0.01).

SNP	CHR	N	Position	kb	*p*_meta_	Variant annotation	Genes within clumping region
rs10093628	8	6	chr8:9393379.9452088	58.71	2.72E−10	Intergenic variant	TNKS
rs7650482	3	4	chr3:12840934.12848822	7.889	5.27E−10	Coding transcript intron variant	CAND2
rs2608280	11	3	chr11:93209472.93264680	55.209	3.60E−09	Downstream gene variant	SMCO4
rs4804414	19	3	chr19:7222655.7223848	1.194	5.98E−09	Coding transcript intron variant	INSR
rs181110840	10	1	chr10:114645185.114645185	0.001	6.00E−09	Intergenic variant	TCF7L2*
rs72682256	14	21	chr14:43069125.43122091	52.967	6.09E−09	Intergenic variant	RP11-90P16.1*
rs8009520	14	20	chr14:103261300.103280657	19.358	6.20E−09	Coding transcript intron variant	TRAF3
rs17412920	22	14	chr22:28628209.28947631	319.423	7.68E−09	Coding transcript intron variant	MIR5739, TTC28
rs7944490	11	20	chr11:17001934.17017622	15.689	8.59E−09	Coding transcript intron variant	PLEKHA7
rs269267	7	1	chr7:140372299.140372299	0.001	9.15E−09	Five prime utr intron variant	DENND2A*
rs7003385	8	4	chr8:41558269.41586773	28.505	1.07E−08	Coding transcript intron variant	ANK1
rs61915371	12	2	chr12:27893972.27896264	2.293	1.14E−08	Coding transcript intron variant	MRPS35
rs62064490	17	12	chr17:9800979.9804724	3.746	1.16E−08	Coding transcript intron variant	RCVRN
rs4841432	8	1	chr8:10583506.10583506	0.001	1.24E−08	Synonymous variant	SOX7
rs2193261	7	2	chr7:117478028.117486934	8.907	1.32E−08	Coding transcript intron variant	CTTNBP2
rs10985975	9	5	chr9:126101008.126123009	22.002	1.44E−08	Intergenic variant	CRB2
rs583887	11	26	chr11:65575263.65663547	88.285	1.52E−08	Upstream gene variant	CCDC85B, CFL1, CTSW, EFEMP2, FIBP, FOSL1, MUS81, SNX32
rs17684514	8	2	chr8:8547642.8574282	26.641	1.59E−08	Intergenic variant	CLDN23
rs1362910	8	2	chr8:30856464.30857668	1.205	2.27E−08	Coding transcript intron variant	PURG
rs12891360	14	3	chr14:104008159.104011429	3.271	2.32E−08	Downstream gene variant	TRMT61A*
rs34954697	2	1	chr2:226918363.226918363	0.001	2.85E−08	Intergenic variant	IRS1*
rs1669801	14	5	chr14:46921092.46936747	15.656	2.98E−08	Intergenic variant	LINC00871
rs2536951	9	1	chr9:126646519.126646519	0.001	3.13E−08	Coding transcript intron variant	DENND1A
rs112583287	6	1	chr6:160919184.160919184	0.001	3.16E−08	Non-coding transcript intron variant	LPAL2
rs4488763	22	1	chr22:32380164.32380164	0.001	3.49E−08	Intergenic variant	YWHAH*
rs117981235	11	1	chr11:9820342.9820342	0.001	3.62E−08	Coding transcript intron variant	SBF2, SBF2-AS1
rs6059938	20	4	chr20:33178324.33187130	8.807	4.18E−08	Coding transcript intron variant	PIGU
rs536445	3	1	chr3:173120103.173120103	0.001	4.30E−08	Five prime utr intron variant	NLGN1
rs117471638	10	1	chr10:93158084.93158084	0.001	4.39E−08	Intergenic variant	LOC100188947
rs1465573	5	1	chr5:157985730.157985730	0.001	4.51E−08	Intergenic variant	EBF1*
rs3735260	7	1	chr7:69064637.69064637	0.001	4.81E−08	Five prime utr exon variant	AUTS2
rs2249850	10	1	chr10:104512006.104512006	0.001	4.87E−08	Coding transcript intron variant	WBP1L


CHR, chromosome; SNP, single-nucleotide polymorphism; T2D, type 2 diabetes.

^*^The nearest gene to this locus.

**Table 3:** Novel shared loci in the cross-trait meta-analysis of smoking status and CAD, MI, and HF (*p*_meta_ < 5 × 10^−^8; single trait *p* < 0.01).

Phenotype	SNP	CHR	N	Position	kb	*p*_meta_	Variant annotation	Genes within clumping region
CAD	rs715694	15	2	chr15:47488977.47489021	0.045	5.07E−09	Five prime utr intron variant	SEMA6D
	rs7868608	9	1	chr9:128746044.128746044	0.001	6.16E−09	Intergenic variant	PBX3*
	rs1603985	3	1	chr3:25148868.25148868	0.001	1.25E−08	Intergenic variant	RARB*
	rs530324	8	1	chr8:27491186.27491186	0.001	1.29E−08	Upstream gene variant	SCARA3*
	rs62263602	3	3	chr3:49991060.50152491	161.432	1.59E−08	Coding transcript intron variant	RBM5, BM5-AS1, BM6
	rs10818125	9	12	chr9:120986288.121008326	22.039	2.29E−08	Intergenic variant	TUBB4BP6*
	rs56399143	4	1	chr4:147630649.147630649	0.001	2.59E−08	Coding transcript intron variant	TTC29
	rs7546040	1	13	chr1:44202991.44247233	44.243	2.77E−08	Coding transcript intron variant	ST3GAL3
	rs6734603	2	1	chr2:182038729.182038729	0.001	2.81E−08	Intergenic variant	ITGA4*
	rs10183073	2	1	chr2:146408408.146408408	0.001	4.07E−08	Intergenic variant	RPL6P5*
	rs2107109	12	1	chr12:113212371.113212371	0.001	4.72E−08	Five prime utr intron variant	RPH3A*
	rs1362727	18	1	chr18:25235351.25235351	0.001	4.84E−08	Intergenic variant	CDH2*
MI	rs62216572	21	2	chr21:46488032.46491155	3.124	5.34E−09	Downstream gene variant	SSR4P1
	rs10490563	2	2	chr2:161914168.161915361	1.194	9.99E−09	Intergenic variant	TANK*
	rs10067365	5	3	chr5:125401016.125432585	31.57	1.47E−08	Intergenic variant	GRAMD3*
	rs530324	8	1	chr8:27491186.27491186	0.001	1.87E−08	Upstream gene variant	SCARA3*
	rs56399143	4	1	chr4:147630649.147630649	0.001	3.17E−08	Coding transcript intron variant	TTC29
	rs288159	5	1	chr5:107364363.107364363	0.001	4.85E−08	Coding transcript intron variant	FBXL17
HF	rs4697140	4	7	chr4:20092322.20114221	21.9	4.03E−08	Intergenic variant	SLIT2*
	rs2680705	17	1	chr17:56495584.56495584	0.001	4.52E−08	Upstream gene variant	RNF43*
	rs6917970	6	3	chr6:129428104.129428850	0.747	4.76E−08	Coding transcript intron variant	LAMA2

CHR, chromosome; SNP, single-nucleotide polymorphism; CAD, coronary artery disease; MI, myocardial infarction; HF, heart failure.

^*^The nearest gene to this locus

**Figure 3:**
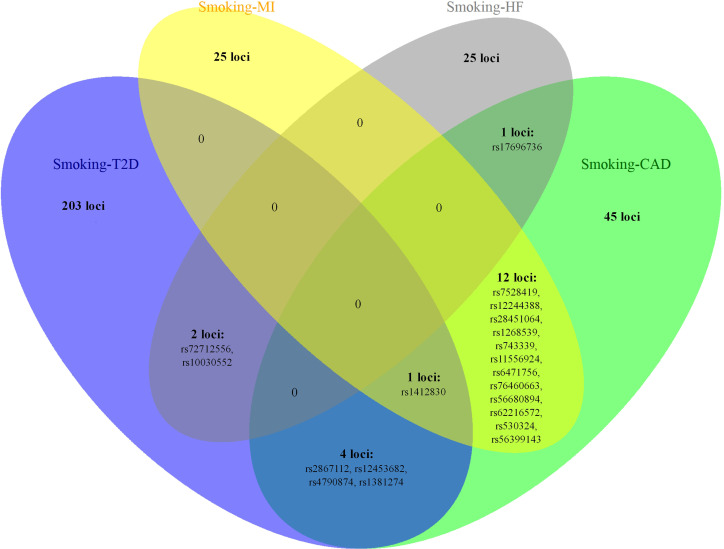
The overlapping loci at the SNP level identified by the cross-trait meta-analysis across different trait pairs. The Venn diagram illustrates the overlapping loci at the SNP level identified by the cross-trait meta-analysis across different trait pairs. T2D, type 2 diabetes; CAD, coronary artery disease; MI, myocardial infarction; HF, heart failure; SNP, single-nucleotide polymorphism.

We found 210 loci significantly associated with both smoking status and T2D, and of these, 32 loci were novel. The most significant locus (index SNP rs9937053, *p*_meta_ = 6.72 × 10^− 81^) was mapped to *FTO* (**Supplementary Table 4**), the first gene contributing to common forms of human obesity (60). Previous studies have indicated that *FTO* is an essential regulator in the development of obesity-induced metabolic and vascular changes (61) and that adiposity-related risk alleles at *FTO* may predispose individuals to diabetes and cardiovascular events (62, 63). A total of 63 genome-wide significant loci were identified in the meta-analysis of smoking status and CAD, of which 12 loci were novel (**Supplementary Table 5**). The most significant locus (index SNP rs1412830, *p*_meta_ = 3.03 × 10^−34^) was mapped to the *CDKN2B-AS1* region, which was also found to be significant in the cross-trait meta-analysis for smoking status and T2D (*p*_meta_ = 2.63 × 10^−17^) or MI (*p*_meta_ = 1.45 × 10^−23^) (**Figure 3**). *CDKN2B-AS1* is a significant genetic susceptibility locus for CVDs and has also been linked to several other pathologies, such as several cancers, T2D, periodontitis, Alzheimer’s disease, and glaucoma (64, 65). A sum of 38 loci, including 6 novel loci, were found to be significantly associated with both smoking status and MI (**Supplementary Table 6**). The top two significant loci (index SNP rs12617922, *p*_meta_ = 4.36 × 10^− 25^; index SNP rs12244388, *p*_meta_ = 7.40 × 10^− 24^) were located at *RPL6P5* and *AS3MT*. *AS3MT* encodes arsenite methyltransferase and plays a role in arsenic metabolism by catalyzing the transfer of a methyl group from *S*-adenosyl-l-methionine (AdoMet) to trivalent arsenical (66). Cigarette smoke contains arsenic with adverse effects and arsenic exposure has been proven to be linked with the risk of acute MI (67). The genome-wide cross-trait meta-analysis between smoking status and HF identified 28 genome-wide significant loci, of which 3 loci were novel (**Supplementary Table 7**). The strongest signal was observed on chromosome 3 at the *CADM2* region (index SNP rs34495106, *p*_meta_ = 3.02 × 10^− 19^), a critical gene associated with a range of behavioral and metabolic traits, including physical activity, alcohol and cannabis use, and obesity (68).

**Figure 4:**
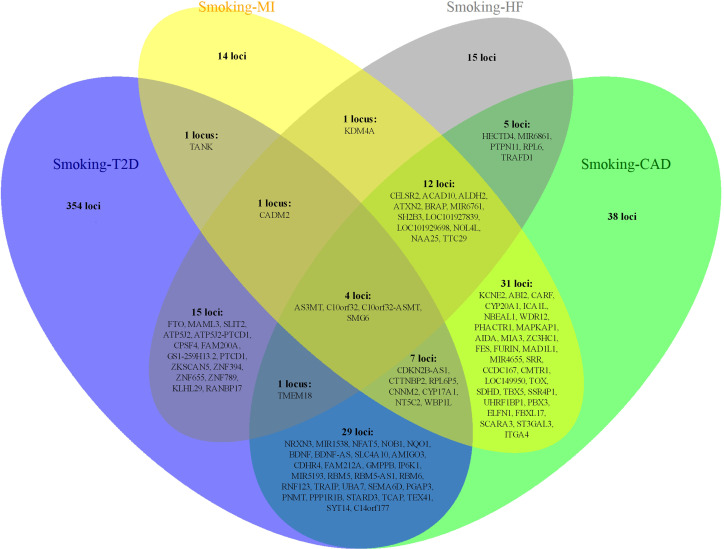
The overlapping loci at the gene level identified by the cross-trait meta-analysis across different trait pairs. The Venn diagram illustrates the overlapping loci at the gene level identified by the cross-trait meta-analysis across different trait pairs. T2D, type 2 diabetes; CAD, coronary artery disease; MI, myocardial infarction; HF, heart failure.

Notably, some shared loci overlapped in the cross-trait meta-analysis of smoking status–T2D and smoking status–CVDs (**Figures 3**, **4**). In addition to the SNP rs1412830 located at the *CDKN2B-AS1* region, we observed four overlapping significant loci (index SNPs: rs12453682, rs1381274, rs2867112, and rs4790874) in the genome-wide cross-trait meta-analysis of smoking status–T2D and smoking status–CAD. Of these, the SNP rs2867112 is near the protein-coding gene body *TMEM18*, and genetic variants in the proximity of the gene have been linked to obesity (69), insulin levels, and blood glucose levels (70). In addition, two loci (index SNPs: rs72712556 and rs10030552) mapped to *MAML3* were found to be genome-wide significant in the meta-analysis of smoking status–T2D and smoking status–HF. These two loci reached genome-wide significance in the single-trait GWAS of smoking status, but their association with T2D or HF remains unknown. More importantly, genes *AS3MT* and *SMG6* were identified in the cross-trait meta-analysis of all four trait pairs (smoking status–T2D, smoking status–CAD, smoking status–MI, and smoking status–HF). Gene *AS3MT* is known to act in arsenic metabolism (66), and polymorphisms in the *AS3MT* have been reported to be associated with CVDs (71) and T2D risks (72, 73). *SMG6* is ubiquitously expressed in many tissues and cell types and has dual functions in telomere maintenance and RNA surveillance pathways (74). Multiple loci in *SMG6* have been proven to be associated with smoking behavior (17) and CAD (75, 76). However, its role in T2D remains to explore.

### Fine-Mapping Credible Set Analysis and Colocalization Analysis

Based on Bayesian fine-mapping, we identified the 99% credible set of causal variants at each of the shared loci. The lists of credible sets of causal variants for each shared locus are provided in **Supplementary Tables 8–11**. In addition, a colocalization analysis was applied to determine whether the two traits were associated and shared the same causal variant at each shared locus. The number of the shared loci considered to colocalize in each trait pair was 20 (smoking status–T2D), 7 (smoking status–CAD), 4 (smoking status–MI), and 4 (smoking status–HF) (**Supplementary Tables 12–15**). Among these, 3 loci (index SNPs: rs329122, rs3742305, and rs1443750) reached a great probability (>95%) of having shared causal variants of smoking status and T2D, in addition to 2 loci (index SNPs: rs11556924 and rs10774625) for smoking status–CAD, 2 loci (index SNPs: rs11556924 and rs653178) for smoking status–MI, and one locus (index SNP: rs4766578) for smoking status–HF.

### Tissue Enrichment Analysis

**Figure 5:**
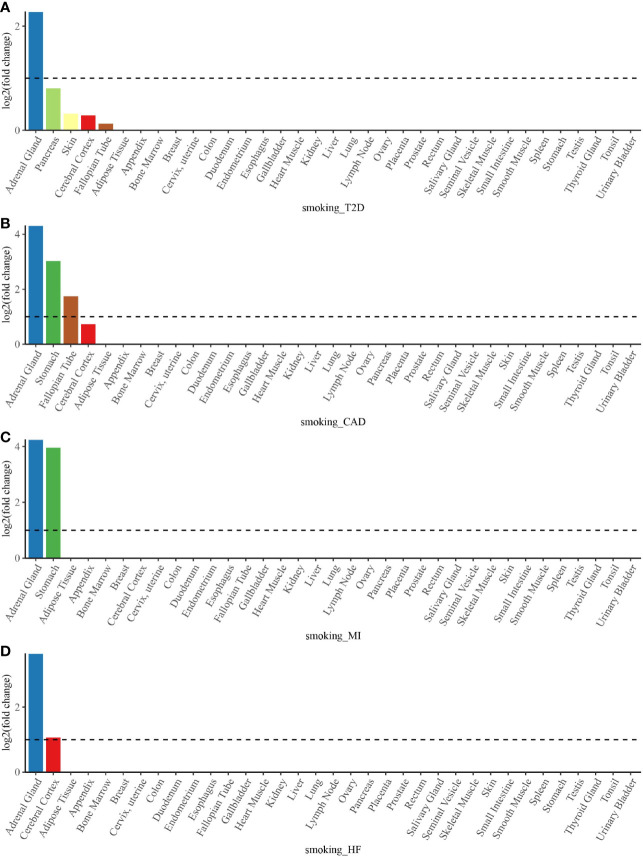
Tissue enrichment analysis for the expression of cross-trait-associated genes between smoking status and T2D **(A)**, CAD **(B)**, MI **(C)**, and HF **(D)**. The vertical axis illustrates the logarithm of tissue expression enrichment fold change based on two. The horizontal axis illustrates 35 independent tissue types. T2D, type 2 diabetes; CAD, coronary artery disease; MI, myocardial infarction; HF, heart failure.

To determine whether shared genes from cross-trait meta-analysis between smoking status and T2D, CAD, MI, and HF were enriched for expression in the disease-relevant tissues, we performed a tissue enrichment analysis using the TissueEnrich web application and tissue-specific genes from RNA-Seq data of the HPA. We found that the shared genes of smoking status with T2D, CAD, MI, and HF were all most strongly enriched in the adrenal gland (**Figure 5**). The stomach was another strongly enriched tissue for the shared genetic genes of smoking status–CAD and smoking status–MI, in addition to the cerebral cortex for the shared genetic genes of smoking status–HF (**Figure 5**).

### Overrepresentation Enrichment Analysis

The overrepresentation enrichment analysis of the GO biological processes highlighted several significantly enriched biological processes for the shared genes between smoking status and T2D, mainly involving regulation of insulin secretion and regulation of peptide hormone secretion (**Supplementary Table 16**). In addition, the shared genes between smoking status and CAD were significantly enriched in the positive regulation of leukocyte adhesion to vascular endothelial cells, axon development, cell morphogenesis involved in neuron differentiation, and neuron projection morphogenesis (**Supplementary Table 17**). However, no significantly enriched biological process for the shared genes of smoking status–MI and smoking status–HF was found.

### Transcriptome-Wide Association Analysis

We conducted a TWAS analysis to explore the genes whose expression in different tissues was associated with smoking status, T2D, CAD, MI, and HF, and to determine if these genes were common among these traits. The lists of gene–tissue pairs significantly associated with each trait are shown in **Supplementary Tables 18–22**. Among these gene–tissue pairs, 354 gene–tissue pairs overlapped between smoking status and T2D, in addition to 37 gene–tissue pairs for smoking status–CAD, 17 gene–tissue pairs for smoking status–MI, and one gene–tissue pair for smoking status–HF (**Supplementary Table 23**). Notably, 17 gene–tissue pairs involving four genes (*FAM117B*, *FES*, *ICA1L*, and *NBEAL1*) for smoking status–MI were contained in gene–tissue pairs for smoking status–CAD, most of which were observed in the nervous, cardiovascular, exo-/endocrine, and digestive systems. *C2orf69*–Brain Caudate basal ganglia gene–trait pair was the only one observed overlapping gene–tissue pair between smoking status and HF. Moreover, the enrichment of smoking status and T2D genes expressed were across multiple tissues, not only including nervous, cardiovascular, exo-/endocrine, and digestive systems but also involving the genital system.

### Mendelian Randomization Analysis

We performed a bidirectional MR analysis to explore the causal relationship between smoking status and T2D, 6 major CVDs, and 8 related metabolic traits. In the detection of the causal effect of smoking status on cardiometabolic traits, we found that smoking status had significant positive causal effects on T2D (β = 0.385, *p* = 3.31 × 10^−3^), CAD (β = 0.670, *p* = 7.86 × 10^−11^), MI (β = 0.725, *p* = 2.32 × 10^−9^), and HF (β = 0.520, *p* = 1.53 × 10^−6^) (**Table 4**). However, the causal effects of smoking status on other traits (IS, ICH, AF, FG, FI, HOMA-B, HOMA-IR, HDL-C, LDL, TC, and TG) were not identified (**Table 4**). In addition, we did not observe any significant causal effect of cardiometabolic traits on smoking status (**Table 4**). Consistent findings that smoking status had significant positive causal effects on T2D, CAD, and HF were observed using additional GWAS data (**Supplementary Table 24**). MR-Egger regression analysis showed that none of the results were affected by horizontal pleiotropy (**Table 4**). These results corroborated each other and supported the robustness of our primary findings.

**Table 4:** Bidirectional MR analysis of smoking status and T2D, CVDs, and related metabolic traits.

Exposure	Outcome	SNPs, n	Inverse variance weighted			MR-Egger		MR-Egger	
			β	*p*-Value	FDR	β	*p*-Value	Intercept	*p*-Value
Smoking status	T2D	127	0.385	3.31E−03	2.48E−02*	−0.022	0.969	0.004	0.470
	CAD	127	0.670	7.86E−11	2.36E−09*	0.195	0.669	0.005	0.286
	MI	127	0.725	2.32E−09	3.48E−08*	0.288	0.596	0.004	0.410
	HF	127	0.520	1.53E−06	1.53E−05*	0.589	0.222	−0.001	0.884
	IS	59	0.573	5.26E−02	1.81E−01	−0.007	0.996	0.005	0.665
	ICH	87	−0.202	7.73E−01	9.41E−01	−2.175	0.469	0.019	0.499
	AF	127	0.018	9.09E−01	9.41E−01	−0.131	0.853	0.001	0.828
	FG	58	0.027	6.65E−01	9.41E−01	−0.240	0.391	0.003	0.328
	FI	58	−0.017	8.02E−01	9.41E−01	0.369	0.224	−0.004	0.193
	HOMA-β	58	0.016	8.07E−01	9.41E−01	0.503	0.082	−0.005	0.084
	HOMA-IR	58	0.014	8.47E−01	9.41E−01	0.328	0.307	−0.003	0.315
	HDL-C	57	−0.145	8.43E−02	2.13E−01	−0.668	0.077	0.005	0.153
	LDL-C	57	0.116	1.95E−01	4.19E−01	0.318	0.430	−0.002	0.606
	TG	57	0.169	5.96E−02	1.81E−01	0.709	0.077	−0.005	0.164
	TC	57	0.158	6.05E−02	1.81E−01	0.203	0.593	0.000	0.903
T2D	Smoking status	202	0.003	2.93E−01	5.17E−01	−0.002	0.727	0.000	0.363
CAD		47	−0.001	9.03E−01	9.41E−01	−0.008	0.522	0.001	0.517
MI		25	0.003	6.40E−01	9.41E−01	−0.024	0.105	0.003	0.048
HF		12	−0.003	8.30E−01	9.41E−01	−0.035	0.546	0.002	0.568
IS^a^		19	0.010	1.11E−01	2.55E−01	−0.029	0.248	0.004	0.110
ICH^a^		13	0.001	7.34E−01	9.41E−01	−0.007	0.457	0.002	0.404
AF		24	0.006	8.52E−02	2.13E−01	0.005	0.571	0.000	0.815
FG		14	0.025	4.97E−02	1.81E−01	0.048	0.108	−0.001	0.361
FI^a^		11	−0.026	2.52E−01	4.72E−01	0.070	0.328	−0.003	0.170
HOMA-β		4	−0.049	2.17E−01	4.34E−01	−0.240	0.222	0.006	0.287
HOMA-IR^a^		13	−0.004	8.38E−01	9.41E−01	0.025	0.735	−0.001	0.678
HDL-C		87	0.000	9.72E−01	9.72E−01	0.004	0.662	0.000	0.619
LDL-C		77	−0.007	5.77E−02	1.81E−01	−0.002	0.636	0.000	0.263
TG		55	−0.001	8.43E−01	9.41E−01	0.004	0.642	0.000	0.463
TC		88	−0.009	1.27E−02	7.61E−02	−0.005	0.415	0.000	0.393

False discovery rate (FDR) Benjamini–Hochberg procedure was used to correct for multiple testing (FDR < 0.05).

MR, Mendelian randomization; SNPs, single-nucleotide polymorphisms; Rg, genetic correlation estimate; SE, standard error of genetic correlation estimate; T2D, type 2 diabetes; CAD, coronary artery disease; MI, myocardial infarction; HF, heart failure; IS, ischemic stroke; ICH, intracerebral hemorrhage; AF, atrial fibrillation; FG, fasting glucose; FI, fasting insulin; HOMA-β, β-cell function obtained by homeostasis model assessment; HOMA-IR, insulin resistance obtained by homeostasis model assessment; HDL-C, high-density lipoprotein cholesterol; LDL-C, low-density lipoprotein cholesterol; TC, total cholesterol; TG, triglyceride.

a Few SNPs achieved genome-wide significance in the original GWAS; in order to obtain valid and reliable instrumental variables for MR analysis, we set the p-value threshold to 1 × 10^−5^.

^*^A significant p-value after Benjamini-Hochberg correction.

## Discussion

To our knowledge, this is the first study to systematically explore shared genetic etiology and the causal relationship between smoking status and T2D and CVDs. First, we found strong positive genetic correlations and further identified shared genetic loci between smoking status and T2D, CAD, MI, and HF. Second, we found that the shared genetic loci were mainly enriched in the adrenal gland and stomach tissues and the biological pathways of nervous system development and regulation of peptide hormone secretion. Third, our TWAS further provided evidence that the enrichment of shared genes expressed was across multiple tissues, including exo-/endocrine, cardiovascular, nervous, digestive, and genital systems. Finally, we identified the causal associations of smoking status with T2D, CAD, MI, and HF. In general, exploration of the shared genetic architecture and causality between smoking status and T2D or CVDs furthers the understanding of the biological mechanisms underlying this comorbidity.

The strong genetic correlations consistent with previous studies (21, 77) suggested that the phenotypic correlations between smoking status and T2D, CAD, MI, and HF were due to a common genetic predisposition base, and we further identified 210 shared genetic loci for smoking status–T2D, in addition to 63 loci for smoking status–CAD, 38 loci for smoking status–MI, and 28 loci for smoking status–HF in the genome-wide cross-trait meta-analysis. Among these shared genetic variants, 32 novel loci were found for smoking status–T2D, along with 12 novel loci for smoking status–CAD, 6 novel loci for smoking status–MI, and 3 novel loci for smoking status–HF, demonstrating the great power of cross-trait meta-analysis in identifying specific shared loci. We highlight several overlapping loci or genes in different trait pairs, which may provide more effective genetic targets for the timely prevention, diagnosis, and treatment of smoking-related T2D and CVDs. The only top locus common to the smoking status–T2D, smoking status–CAD, and smoking status–MI meta-analysis was rs1412830 mapped to *CDKN2B-AS1*. *CDKN2B-AS1* gene is an indispensable long non-coding RNA in multiple diseases (65). In addition to T2D and CVDs (64), *CDKN2B-AS1* has been shown to be aberrantly expressed in various malignancies, idiopathic pulmonary fibrosis, endometriosis, inflammatory bowel disease, and primary open-angle glaucoma and to participate in the progression of lipids, carbohydrate metabolism, and inflammation regulation (65), which is likely to serve as a promising therapeutic target or prognostic biomarker in multiple human diseases. The SNP rs2867112 near the protein-coding gene body *TMEM18* was found to be significant in the meta-analysis for smoking status–T2D and smoking status–CAD. *TMEM18* is an important susceptibility locus for obesity (69), which is an independent risk factor for the development and progression of T2D and CVDs. A previous study provided evidence that smoking might modify the genetic effects of *TMEM18* on body mass index (BMI), a proxy for adiposity (78). In addition, two loci (index SNPs: rs72712556 and rs10030552) mapped to *MAML3* were found to have genome-wide significance in the meta-analysis of smoking status–T2D and smoking status–HF, which reached genome-wide significance in the single-trait GWAS of smoking status, but its association with HF or T2D remains unknown and deserves in-depth study. *AS3MT* and *SMG6* are two important genes that were identified in the cross-trait meta-analysis of all four trait pairs (smoking status–T2D, smoking status–CAD, smoking status–MI, and smoking status–HF). Cigarette smoke is a vital source of ingested low-level arsenic, and chronic arsenic exposure is associated with increased morbidity and mortality from CVDs (71, 79) and an increased risk of T2D (72, 73). Polymorphisms in *AS3MT* gene are associated with the efficiency of arsenic biotransformation (66, 72), suggesting that the mechanisms of arsenic metabolism and biotransformation may play an important role in smoking-related T2D and CVDs. Multiple loci in *SMG6* have been proven to be associated with smoking behavior (17) and CAD (75, 76). Moreover, a previous study has shown that tobacco smoking is associated with the methylation of genes related to CAD, which includes *SMG6* gene (75). These findings provide novel insights into the pathways that link tobacco smoking to the risk of CVDs. However, the role of *SMG6* gene in smoking-related T2D remains to be explored.

In addition to the significant findings in the shared genes related to both smoking and T2D or CVDs, we identified the relevant tissues and biological processes that the shared genes enriched in which suggests the potential biological mechanisms that confer comorbid effects. Tissue enrichment analysis showed that the shared genes of smoking status with T2D, CAD, MI, and HF were all most strongly enriched in the adrenal gland. A previous study has reported that cigarette smoking is a strong activator of the hypothalamus–pituitary–adrenal (HPA) axis followed by significant elevations in the adrenal hormone cortisol (80). Cortisol plays an important role in lipid and glucose metabolism; and elevated cortisol levels, if prolonged, lead to a redistribution of body fat characterized by truncal obesity, which is a risk factor for T2D and CVDs (81). Activation of the HPA axis is also thought to contribute to drug abuse during the addictive process, which may also contribute to the abuse-related effects of cigarette smoking (82). In the overrepresentation enrichment analysis, the biological pathway of insulin secretion was found to be significant for the shared genes of smoking status and T2D, indicating that smoking can affect pancreatic islet cell function. Many studies have found neuronal nicotinic acetylcholine receptors (nAChRs) expressed on pancreatic islet cells (83), and these functional nAChRs sensitive to nicotine in pancreatic cells may be a switch to modulate pancreatic cell physiological function and involved in tobacco toxicity (84). Furthermore, several studies in animal models have shown that nicotine can increase apoptosis of islet β-cells, thus reducing insulin secretion (85–88). Mitochondrial dysfunction, oxidative stress, and inflammation are involved as underlying mechanisms for the direct toxicity induced by nicotine *via* nAChRs (84). The stomach was another strongly enriched tissue for the shared genetic loci of smoking status–CAD and smoking status–MI. Relevant studies have shown that smoking can increase the probability of getting heartburn and peptic ulcers (89), and gastrointestinal diseases may trigger myocardial ischemia-related chest pain probably through the afferent vagal fibers shared by the esophagus and the heart to induce a coronary spasm (90, 91). In addition, the shared genes for smoking status–HF/CAD were enriched in cerebral cortex tissue and the biological pathways of nervous system development, indicating the important role of the nervous system on the comorbidity of smoking and CVDs. Nicotine and fine particulate matter in tobacco smoke can lead to increased sympathetic nerve activity (92), which is one of the hallmarks of chronic congestive HF (93) and plays a role in the process of atherosclerosis (94).

Our TWAS further provided evidence that the shared genes were mostly from the exo-/endocrine, cardiovascular, nervous, and digestive systems. In addition, the TWAS result reported the enrichment of the shared genes between smoking status and T2D from the genital system. Smoking and T2D have a variety of adverse effects on the genital system (95, 96). More importantly, smoking and diabetes may influence the epigenetic modification during the production of germ cells, and these epigenetic dysregulations may be inherited through the germ line and passed onto more than one generation, which in turn may increase the risk of related diseases in offspring (97). A total of 58 significant genes in TWAS were also found to be genome-wide significant in cross-trait meta-analysis for smoking status–T2D, in addition to 13 genes for smoking status–CAD and 3 genes for smoking status–MI, which further indicated the fact that a significant portion of shared genetic loci we identified in the cross-trait meta-analysis were indeed functional variants of modulating gene expression on influencing both phenotypes. Among these, we highlight the importance of the gene *TCF7L2*, which showed significance in the cross-trait meta-analysis and TWAS of smoking status and T2D. SNPs in *TCF7L2* are especially known to be associated with a higher risk of developing T2D (98). Recently, a study has suggested that *TCF7L2* links nicotine addiction to diabetes in animal models. This study has revealed that *TCF7L2* is densely expressed in the medial habenula and plays an important role in regulating the function of nAChRs in the habenula and in controlling nicotine intake (22). Habenular neurons provide polysynaptic input to the pancreas, and nicotine acts on this habenula–pancreas circuit, in a *TCF7L2*-dependent manner and *via* the autonomic nervous system, to increase blood glucose levels (22). Furthermore, *FES*, *ICA1L*, and *NBEAL1* genes showed significance in the cross-trait meta-analysis and TWAS of smoking status–CAD and smoking status–MI and expressed in multiple tissues, including the brain, nerve, artery, adipose, pancreas, and thyroid tissues. Gene *FES*, which encodes the human cellular counterpart of a feline sarcoma retrovirus protein with transforming capabilities, is well known to be associated with myeloid leukemia (99), but recent studies observed the function of *FES* in modulating atherosclerotic plaque vulnerability (100) and the effect of tobacco smoking on DNA methylation of *FES* (75). Genes *ICA1L* and *NBEAL1* were mapped by the same locus (index SNP: rs114123510), and both are related to cholesterol metabolism, in which dysregulation promotes the pathology of atherosclerosis, MI, and strokes (101). Notably, *C2orf69*–Brain Caudate basal ganglia gene–trait pair was the only one observed overlapping gene–tissue pair between smoking status and HF. *C2orf69* is an evolutionarily conserved gene whose function needs to be further clarified, but recent studies have shown its association with a fatal autoinflammatory syndrome that disrupts the development/homeostasis of the immune and central nervous systems (102, 103), which may contribute to the link between smoking and HF.

In addition to pleiotropy, the associations between smoking status and these cardiometabolic traits may be due to causality. Consistent with previous large cohort (4, 5) and MR studies (104–106), our exploratory bidirectional MR analysis found that smoking status had significant positive causal effects on T2D, CAD, MI, and HF, which suggests that the genetic correlations of the above trait pairs are attributed to both shared genetic architecture and causality. However, we did not observe a significant causal association between smoking status and IS, which is inconsistent with two recent studies (104, 105). This may be due to the different definitions of smoking, involving different ancestry populations, and different sample sizes, which need further confirmation. Besides, we did not observe any causality in the detection of the causal effect of 15 cardiometabolic traits on smoking status, excluding the possibility of reverse causation between smoking status and T2D or CVDs. The potential mechanisms underlying the causal relationship between smoking and T2D or CVDs require further investigation, but the shared loci and related pathways could provide new insights and directions.

In addition, we explored the genetic correlations between smoking status and T2D/CVD-related metabolic traits and observed a nominal positive correlation of smoking status with FG, a weak negative correlation of smoking status with HDL-C, and a weak positive correlation of smoking status with TG. Lipid and glycemic traits, resulting from complex and interwoven physiological mechanisms, are indicators of T2D and CVD risks, and understanding their associations with smoking can provide better insight into the pathophysiological intersect of T2D and CVDs. Previous studies have proven the role of smoking in elevating plasma TG concentration, decreasing plasma HDL-C concentration (107), and increasing the risk of impaired FG (108) and insulin resistance (109), which enhance the increased risk of T2D and CVDs. Although smoking cessation can ameliorate these changes, it is worth noting that smoking cessation is frequently followed by weight gain, which can contribute to the increased short-term risk of T2D (5, 110). Therefore, for smokers at risk for T2D, smoking cessation should be coupled with strategies for T2D prevention and early detection (5).

We acknowledge the limitations of our study. Despite the large sample sizes and high power of the GWAS summary statistics coming from meta-analysis studies, the homogeneity among different summary statistics was reduced. However, each study conducted study-specific quality control to ensure data quality. In addition, simulations have confirmed that the effect of population structure and cryptic relatedness could be controlled well by our cross-trait meta-analysis method CPASSOC. Second, because of the concerns on sample size, accuracy, and availability of the GWAS data, we only analyzed smoking status in this study and did not consider quantitative smoking phenotypes such as cigarettes smoked per day or the years of smoking. Besides, smokeless tobacco products such as snuff tend to show different associations with T2D or CVDs as compared to cigarette smoking (111–113). It is important to consider these phenotypes in future investigations to shed light on the relationship between smoking and T2D or CVDs. Third, limited to the existing original GWASs, the sample sizes of some original trait-specific GWASs, especially ICH, were relatively small, which resulted in limited statistical power (**Supplementary Table 25**). Fourth, to yield reliable results, we used the data from the largest or latest GWASs, but there may be sample overlap between smoking status and T2D, CAD, and HF, which can influence the inference of causality in MR analysis. However, we used additional GWAS data of these traits with no sample overlap with smoking status GWAS to further confirm our primary findings and observed highly consistent results. Such consistency reinforced the robustness of our findings. Fifth, additional appropriate data were not available for us to replicate our findings. However, we used the data from the largest or latest GWASs for these traits to yield reliable results, and if possible, we will perform replication analysis in the future. Finally, our study was limited to assessing the shared genetic etiology between smoking status and T2D or CVDs. The effects of environmental factors and gene–environment interactions between smoking status and T2D or CVDs still need to be explored in further studies.

In summary, our findings provide strong evidence on shared genetic etiology and causal associations between smoking status and T2D or CVDs, underscoring the potential shared biological mechanisms underlying the link between smoking and T2D or CVDs. This work is important and opens up a new way for more effective and timely prevention, diagnosis, and treatment of smoking-related T2D or CVDs.

## Data Availability Statement

The datasets presented in this study can be found in online repositories. The names of the repository/repositories and accession number(s) can be found in the article/**Supplementary Material**. The download links for all the data relevant to the study can be found in the **Supplementary Material**.

## Ethics Statement

The studies involving human participants were reviewed and approved by the relevant institutional review boards. The patients/participants provided their written informed consent to participate in this study.

## Author Contributions

YC, XW, JJ, and TH designed the research. JJ and TH had full access to all the data in the study and take responsibility for the integrity of the data and the accuracy of the data analysis. YC and XW wrote the paper and performed the data analysis. All authors contributed to the statistical analysis, critically reviewed the manuscript during the writing process, and approved the final version to be published. YC, XW, JJ, and TH are the guarantors for the study.

## Funding

The study was supported by grants from the National Key R&D Program of China (2019YFC2003400), the Peking University Start-up Grant (BMU2018YJ002), and the high-performance Computing Platform of Peking University. The funding organization had no role in the preparation of the manuscript.

## Conflict of Interest

The authors declare that the research was conducted in the absence of any commercial or financial relationships that could be construed as a potential conflict of interest.

## Publisher’s Note

All claims expressed in this article are solely those of the authors and do not necessarily represent those of their affiliated organizations, or those of the publisher, the editors and the reviewers. Any product that may be evaluated in this article, or claim that may be made by its manufacturer, is not guaranteed or endorsed by the publisher.
